# Selection for CD26^−^ and CD49A^+^ Cells From Pluripotent Stem Cells-Derived Islet-Like Clusters Improves Therapeutic Activity in Diabetic Mice

**DOI:** 10.3389/fendo.2021.635405

**Published:** 2021-05-05

**Authors:** Kfir Molakandov, Denise A. Berti, Avital Beck, Ofer Elhanani, Michael D. Walker, Yoav Soen, Karina Yavriyants, Michal Zimerman, Ella Volman, Itzik Toledo, Anna Erukhimovich, Alon M. Levy, Arik Hasson, Joseph Itskovitz-Eldor, Judith Chebath, Michel Revel

**Affiliations:** ^1^ Kadimastem Ltd., Weizmann Science Park, Ness Ziona, Israel; ^2^ Department of Biomolecular Sciences, Weizmann Institute of Science, Rehovot, Israel; ^3^ Department of Molecular Genetics (emeritus), Weizmann Institute of Science, Rehovot, Israel

**Keywords:** human ESC-derived insulin producing cells, islet-like clusters (ILC), functional cell capture screening, integrin alpha1 (CD49A), DPP4 (CD26), alginate encapsulation, STZ-treated C57BL/6 mice diabetes models

## Abstract

**Background:**

Cell therapy of diabetes aims at restoring the physiological control of blood glucose by transplantation of functional pancreatic islet cells. A potentially unlimited source of cells for such transplantations would be islet cells derived from an *in vitro* differentiation of human pluripotent stem cells (hESC/hiPSC). The islet-like clusters (ILC) produced by the known differentiation protocols contain various cell populations. Among these, the β-cells that express both insulin and the transcription factor Nkx6.1 seem to be the most efficient to restore normoglycemia in diabetes animal models. Our aim was to find markers allowing selection of these efficient cells.

**Methods:**

Functional Cell-Capture Screening (FCCS) was used to identify markers that preferentially capture the cells expressing both insulin and Nkx6.1, from hESC-derived ILC cells. In order to test whether selection for such markers could improve cell therapy in diabetic mouse models, we used ILC produced from a clinical-grade line of hESC by a refined differentiation protocol adapted to up-scalable bioreactors. Re-aggregated MACS sorted cells were encapsulated in microspheres made of alginate modified to reduce foreign body reaction. Implantation was done intraperitoneally in STZ-treated C57BL/6 immuno-competent mice.

**Results:**

CD49A (integrin alpha1) was identified by FCCS as a marker for cells that express insulin (or C-peptide) as well as Nkx6.1 in ILC derived by hESC differentiation. The ILC fraction enriched in CD49A**^+^** cells rapidly reduced glycemia when implanted in diabetic mice, whereas mice receiving the CD49A depleted population remained highly diabetic. CD49A-enriched ILC cells also produced higher levels of human C-peptide in the blood of transplanted mice. However, the difference between CD49A-enriched and total ILC cells remained small. Another marker, CD26 (DPP4), was identified by FCCS as binding insulin-expressing cells which are Nkx6.1 negative. Depletion of CD26**^+^** cells followed by enrichment for CD49A**^+^** cells increased insulin^+^/Nkx6.1^+^ cells fraction to ~70%. The CD26**^-^**/CD49A**^+^** enriched ILC exhibited improved function over non-sorted ILC or CD49A**^+^** cells in diabetic mice and maintain prolonged blood C-peptide levels.

**Conclusions:**

Refining the composition of ILC differentiated from hPSC by negative selection to remove cells expressing CD26 and positive selection for CD49A expressing cells could enable more effective cell therapy of diabetes.

## Introduction

Diabetic conditions due to destruction (in type I) or dysfunction (in type II) of pancreatic islets of Langerhans, have detrimental impacts on the quality of life and lifespan. For the hundred million patients depending on frequent insulin injections for controlling blood glucose levels, islet cells transplantation would have the advantage to restore a physiological regulation of glycemia. The Edmonton protocol (1–3) based on transplantation through the portal vein of human islets obtained from brain dead donors has allowed patients to become insulin-free for significant periods of time. Yet, the availability of such islet donations is too limited to meet the transplantation demand. An alternative, more abundant supply may be generated by differentiation of islet-like clusters (ILC) from large-scale cultures of human pluripotent cells (hPSC). Recently developed multistage protocols of differentiation produce pancreatic islet cell populations including mature β-cells that function in reducing glycemia in animal models of diabetes (4–6). New methods for microencapsulation in modified alginate support long term function after implantation in diabetic animals (7–9).

A drawback is that *in vitro* differentiated ILC contain additional populations of cells, which may not be necessary for islet function, and even may impair the efficacy or the safety of the transplanted cells for future clinical treatment of diabetes. To alleviate this problem, we investigated which cell surface marker could help identify and isolate the ILC cells that have higher capacity to normalize glycemia in diabetes models. We screened a large array of antibodies to cell-surface proteins by the Functional Cell-Capture Screening (FCCS) previously developed to identify cell-surface markers selective for endoderm and non-endoderm populations of differentiating hESC (10). The same procedure was later used to characterize surface markers of cells in post-mortem islets from adult humans (11). The present study was aimed to generate more potent ILC by using antibodies that were identified by the FCCS as selecting for insulin-producing cells in ILC differentiated from human ESC. Starting from a clinical-grade hESC line, we refined a 30-day protocol, in spinner suspension cultures or controlled bioreactors, which converts 3D clusters of these highly pluripotent stem cells into pancreatic islet-like clusters. The clusters were dissociated into single cells and fractionated according to their capacity to bind, or not bind, to two antibodies found by FCCS to capture insulin-producing cells. One antibody against integrin-alpha1 (CD49A) was found to bind the insulin-producing cells that express Nkx6.1, a transcription factor essential for the formation and function of mature β-cell s (12, 13), but to bind also insulin-producing cells lacking Nkx6.1. Another antibody, against Dipeptidyl peptidase-4 (CD26), was found to bind only insulin-producing cells that do not express Nkx6.1. A novel sorting strategy is suggested by first removing cells expressing CD26 and then enriching for CD49A positive cells. Thus, it is possible to increase the *in vivo* therapeutic activity of ILC to normalize glycemia in diabetic mice by two consecutive selections and supports the feasibility of functional enrichment strategies to improve the activity of hPSC-derived ILC for the treatment of diabetes.

## Methods

### Human ES Cell Expansion and Differentiation to Islet-Like Clusters

Highly pluripotent, clinical grade, human ES cells HADC-100 (14) (provided by Professor Benjamin Reubinoff, Hadassah Medical School, Jerusalem, Israel) were grown to confluent monolayers in essential E8 medium (Gibco, Cat#A1517001), with addition of penicillin and streptomycin (PS, Gibco, Cat#15140-122) on vitronectin-coated flasks (Gibco, Cat# A14700). Differentiation was performed on cell aggregates formed in spinner flasks during 2 days in dynamic suspension cultures. In brief, 48 h before starting the differentiation protocol (day-2), non-differentiated cells were dissociated with Versene (Gibco, Cat#15040033). Single cells washed with PBS^−/−^ (Gibco, Cat#14190-094), were seeded in 500 ml disposable spinner flasks (Corning, Cat#CZ-3153), filled with 250 ml E8 medium containing 10 µM Rock Inhibitor Y27632 (Cayman Chemical, #10005583-10), at concentration of 0.8–1 × 10^6^ cells/ml. The spinner flasks were placed on magnetic stirrer (DURA-MAG, 9 position stirrer, Chemglass) at speed of 70 rpm in a humidified incubator set at 5% CO_2_ and 37°C. This resulted in the formation of ES cell clusters in suspension, as well as in cell proliferation. On day -1, 80% of the E8 medium was replaced. On Day 0, the E8 medium was washed away (15) by letting the aggregates settle for 5 min and removing the supernatant with a pipette. Cells were washed with 250 ml PBS^−/−^; after 3 min stirring in the incubator, PBS^−/−^ was replaced by 250 ml of stage 1 differentiation medium. The media for the seven-stage differentiation protocol, refined on the basis of several published protocols (5, 16–19), are detailed in additional files 1 and 2 (**Tables S1** and **S2**).

For Bioreactors, the hESC aggregation and differentiation was similarly done in the DASbox® mini system (Eppendorf) with online monitoring of culture parameters. Up to four parallel bottles containing 150 ml medium were seeded with 0.8–1 × 10^6^ cells/ml 48 h before and washed with PBS just before differentiation as above. Medium changes were done batch-wise in the semi-closed system using peristaltic pumps.

### Real-Time Quantitative PCR

RNA was isolated from cells using the RNAeasy micro kit (Qiagen #74004) and purified from genomic DNA with RNase-free DNase kit (Qiagen #79254). The cDNA synthesis was done with the high-capacity cDNA Reverse Transcription kit (Applied Biosystems 4368814). Transcript levels were measured by real-time qPCR using Taqman Fast advanced master mix (Applied Biosystems #4444557). The level of each gene was normalized to endogenous HPRT gene, using the 2^−ΔΔ^ *CT* method. The probes used for qPCR are listed in **Table S3**. The MARIS procedure (20) is described in Additional file 4.

### Flow Cytometry

Samples from settled aggregates during or at the end of the differentiation process were dissociated with Accumax (Sigma, Cat# A7089) at 0.35 ml per 300,000 cells for 8–10 min, after which the enzyme was blocked in 1 ml PBS^−/−^ with 10% FBS, and the cells were centrifuged (350 × g, 3 min). For external cell membrane labeling, cells were washed in PBS^−/−^, and antibodies (e.g. anti-CD49A, as listed in **Table S4**) were directly added to the cell suspension in FACS buffer (0.5% BSA in PBS) followed by incubation at 4°C for 30 min. For internal antigen labeling (e.g. anti-human C-peptide and Nkx6.1, **Table S4**), cells after centrifugation were washed once in 1 ml PBS and fixed in 0.4 ml of 4% paraformaldehyde (PFA, EMS Cat# 15710), for 20 min at 4°C. After two washes with PBS cells were incubated for 1 h at 4°C in blocking solution [PBS with 5% Bovine serum albumin (BSA) and 3% horse serum] containing 0.3% Triton X-100 (Sigma, T6878), and washed once with PBS. The cell pellet suspended in 0.1 ml blocking solution (with 0.1% Triton x-100) containing antibodies, was incubated overnight at 4°C, or for 1 h at room temperature (RT), and washed with PBS. When fluorescent tag-conjugated primary antibodies were used, fluorescence was read after this step in Flow Cytometer-BD FACS Canto II. Otherwise, cells were incubated in blocking solution containing 1:100 dilutions of the fluorescent tag-conjugated secondary antibody and washed with PBS before reading fluorescence.

### Functional Cell Capture Screening (FCCS) on Antibody Arrays

Antibody arrays were printed in a Microgrid printer with solid pins (Total array Systems, BioRobotics, Cambridge, UK) on hydrogel-coated slides (Full Moon Biosystems, Sunnyvale, CA, USA) using a panel of 235 monoclonal mouse anti-human antibodies (BD biosciences), each antibody being spotted at five different places in the array, as described before (10, 11) (US patent 2018/0369290 A1, Item 0081). The cell clusters were dissociated using TrypLE Express (Invitrogen Cat#12604) for 4 min, followed by quenching with 10% FBS in PBS, centrifugation, and resuspension in CMRL. The printed area of the array was blocked for 3 min with 1% BSA in PBS solution, before cell seeding, at about 0.5 × 10^6^ cells/ml in 0.25–0.5 ml of CMRL medium, supplemented with 2 µl of DNase I (Ambion 2 U/µl) and incubation was for 1 h at 37°C. Excess cells were removed in a large volume of PBS and the arrays were fixed in 4% PFA for 10 min. Cells on the array were permeabilized in PBS, 0.2% Triton X-100 for 20 min, washed twice with PBS, and blocked for 45 min in blocking buffer (2% FBS, 2% BSA, 50 mM glycine in PBS). After blocking, arrays were washed twice with PBS and incubated for 2 h at RT in blocking buffer with 0.1% of Triton X-100 containing the primary antibody guinea-pig anti-insulin (DAKO, A0564). Primary antibodies were removed, and arrays were washed three times with working buffer. Then, secondary antibodies were added in working buffer for 45 min at room temp: cy5 donkey anti-guinea–pig (Jackson ImmunoResearch 706-175 -148). Arrays were washed three times in working buffer and imaged using automated, high content fluorescence microscopy (IXmicro, MDC). Total cells in each spot were counted by phase microscopy and the percent of insulin positive cells was calculated. Three repeats were performed with different batches of ES-derived cells at stage 7 of the differentiation protocol. The significance of the amount of cell binding to surface antibody was evaluated by two sample paired T-test (*P* value less than 0.05). In another set of experiments, cells were reacted with antibodies against insulin as above but also with antibodies against Pdx1 and against Nkx6.1 (see **Table S4**). The number of cells stained for PDX1 and Insulin, and for insulin and Nkx6.1 was counted.

### Magnetic Activated Cell Sorting (MACS)

Cell clusters after stage 7 of differentiation were washed in PBS^−/−^, dissociated with Accumax (10 ml for 25 × 10^6^ cells) for 10 min at 37°C and washed with CMRL 2% BSA. Dissociated cell suspension was passed through a 30 µm MACS filter previously washed with PBS^−/−^ and counted using Nucleocounter^®^ NC-200. Part of the clusters was set aside for control (non-dissociated, non-selected cells), seeded at 10^6^ cells per ml in ultra-low binding six-well-plates (Corning Cat#CLS3471) and left in the incubator on orbital shaker (NovaShake-B32X) set at 95 rpm for 3 days in medium CMRL^+^ (**Table S2**), before implantation. Cells were suspended in MACS buffer (PBS^−/−^, 2% BSA, 2 mM EDTA, sterile, degassed), 100 µl per 10^7^ cells, for reaction with anti-CD49A-PE (Myltenyi, cat# 130-101-397) using 10 µl per 10^7^ cells for 10 min at 4°C, followed by washing with 5 ml MACS buffer. Cells suspended in MACS buffer (80 µl per 10^7^ cells) were reacted with 20 µl per 10^7^ cells of anti-PE magnetic microbeads (Miltenyi; cat# 5181214192) for 15 min at 4°C. After washing with 5 ml cold MACS buffer and centrifugation, cells were suspended in MACS buffer and applied to pre-separation filters and LS MACS column(s) as recommended by the manufacturer. Prior to implantation, all single cells fractions were re-aggregated in suspension in non-TC treated six-well plates (Corning Cat#CLS3471-24EA) in CMRL^+^, 10 µM RI Y27632 and 2 µg/ml Laminin (Bio Lamina Cat#MX521CTG), on orbital shaker.

For removal of CD26 positive cells prior to CD49A enrichment, the cells were dissociated as above, incubated for 10 min at 4°C with anti-CD26-PE (cat#302706), (10 µl/10^7^ cells, in 100 µl MACS buffer for 10^7^ cells), and after washing, reacted with anti-PE microbeads as described above. The mixture, after washing and resuspension in MACS buffer, was applied to LS column(s) and the flow through fraction (CD26 depleted) kept for further fractionation by MACS with anti-CD49A antibody.

### Diabetes Induction in Mice and ILC Implantation

Six-to-8-week-old immune-competent mice C57BL/6JOlaHsd (Harlan, Israel) were rendered diabetic by intraperitoneal injection of streptozotocin (STZ) (Sigma, Cat#S0130), using 4 × 50 mg STZ/kg after 6 h daily fasting. Implantation was performed in diabetic mice, defined by blood glucose higher than 250 mg/dl for three consecutive tests. Blood glucose was measured by a glucometer, on tail vein blood. Intraperitoneal Glucose Tolerance Test (IPGTT) was done after fasting the mice overnight by i.p. injection of glucose (2 g/kg). Blood glucose was then monitored during a 2 h period.

For implantation of micro-encapsulated ILC cells (see below), mice were anesthetized by an IP injection of ketamine/xylazine (Sigma, K4138) at 87.5 mg/kg ketamine/12.5 mg/kg xylazine and then mounted on a surgical pad. The skin was prepared by shaving with electric clippers, application of Polydin, and then 70% ethanol solution. An abdominal incision (1 cm), and peritoneal incision (0.5 cm) allowed to insert microencapsulated ILC into the peritoneal cavity of the mouse using a 1 ml sterile plastic tip (about 0.5 ml total volume). The peritoneum and the skin were closed with sutures and cleaned with Polydin. The mice were kept warm by a heating pad till they woke up. The cell doses implanted were between 1.0 and 2 × 10^6^ cells, as indicated.

### Human C-Peptide ELISA Assays

The levels of human C-peptide, which reflect the levels of insulin secreted by the human ILC, were measured in blood samples collected after anesthesia from mouse retroorbital sinus. To test the insulin/C-peptide response to glucose, mice were withdrawn from food for 12 h, and injected intra-peritoneally with a glucose solution (25%, 41-302-500, Biological Industries). Blood was collected before and 30 min after the glucose injection, centrifuged, and stored at −20°C. ELISA assays were performed using ultra-sensitive ELISA kit for human C-peptide from Mercodia (#10-1141-01) according to instructions.

### Preparation of TMTD-Modified Alginate and Microcapsules

Triazole thiomorpholine dioxide (TMTD Y1-Z15) preparation and coupling to alginate PRONOVA UP-MVG alginate (NovaMatrix) were done for Kadimastem, by Recipharm-Israel as described [8, 9]. After verification of the product structure by NMR, purification by filtration, dialysis, and desiccation, elemental analysis revealed that more than 50% alginate guluronic or mannuronic residues were coupled to TMTD. Solutions of 4.6% of TMTD-coupled alginate were used [80% in volume of 5% (w/v) TMTD-coupled UP MVG and 20% of 3% (w/v) UP MVG]. Stage 7 ILC (see **Table S1**), washed with KREBS buffer without Ca^++^ pH 7.4, were mixed with alginate solutions at the concentration of about 10^7^ cells/ml in a 5 ml Eppendorf tube. The micro-encapsulator Buchi B395, located in a tissue culture laminar flow hood for sterility, was set up to obtain microcapsules of 1.5 mm diameter with 4.6% alginate polymerized in CaCl_2_ (100 mM in HEPES pH 7.4).

### ILC Immunostaining and Imaging

Stage 7 cell clusters were fixed in 4% PFA and washed in PBS^−/−^. A minimum volume of warm 1% agarose was added to the pellet of clusters. After agarose became solid, the block was embedded in paraffin, and 10 µm thick sections were produced and bound to glass slides. After removal of paraffin by alternative baths of xylene and ethanol, antigen retrieval was performed by heating slides in 10 mM citrate buffer pH 6.0 (ZYTOMED systems), for 15 min in pressure cooker (Bio TintoRetriever). Blocking and permeabilization was done by incubation with PBS containing 5% BSA, 3% horse serum (blocking solution) supplemented with 0.3% Triton X-100 for 1 h at RT. Antibodies against PDX1, C-peptide, Nkx6.1 (**Table S4**) were diluted blocking solution supplemented with 0.1% Triton X-100 and incubated overnight at 4°C in humidified chambers. After two washes in PBS, incubation with secondary antibodies was done for 1 h at room temperature and washes were done similarly. Nuclei were stained with DAPI (1 µg/ml). The slides were mounted with aqueous mounting medium and covered with coverslip. Images were obtained using Nikon Eclipse 80i fluorescence microscope.

## Results

### Islet-Like Clusters Differentiated From hESC Contain Mature β-Cells

**Figure 1:**
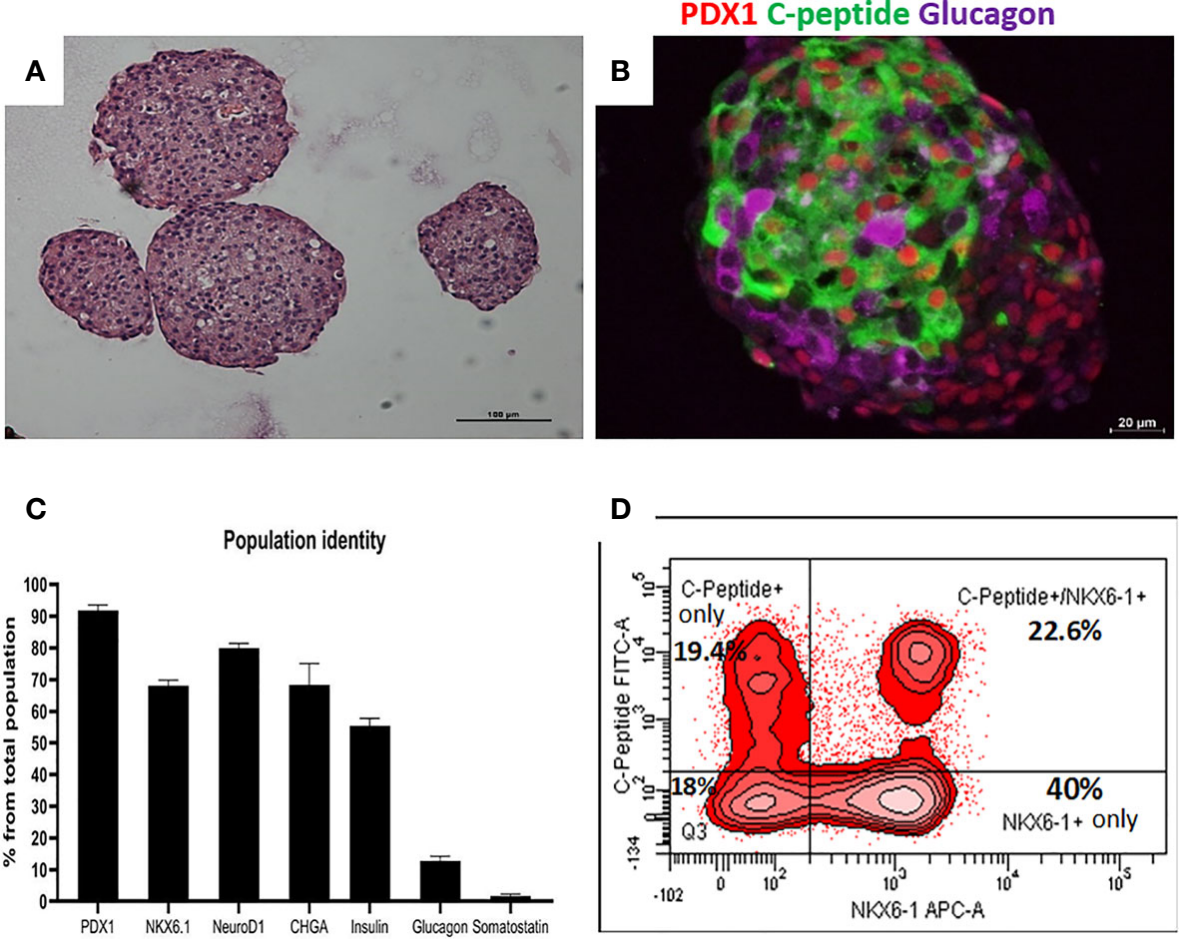
Cell populations identity in Islet-like clusters at the end of the differentiation process. **(A)** H&E of microencapsulated ILC, demonstrating clusters ranging from 100 to 200 µm. **(B)** Immunostaining of an ILC for human C-peptide (green), Glucagon (purple), PDX1 (red, nuclear stain). **(C)** Percentage of ILC cells stained by antibodies to indicated proteins, calculated from flow cytometry analysis of a representative ILC preparations (n = 10, different batches, antibodies listed in **Table S4**). **(D)** Flow-cytometry analysis of dissociated total ILC cells, fixed and stained for human C-peptide and Nkx6.1. The percentage of cells with C-peptide only (no Nkx6.1), with C-peptide and Nkx6.1, with Nkx6.1 only (no C-peptide) is shown. Q3 is the double negative fraction; C-peptide**^−^**/Nkx6.1**^−^**.

**Figure 2:**
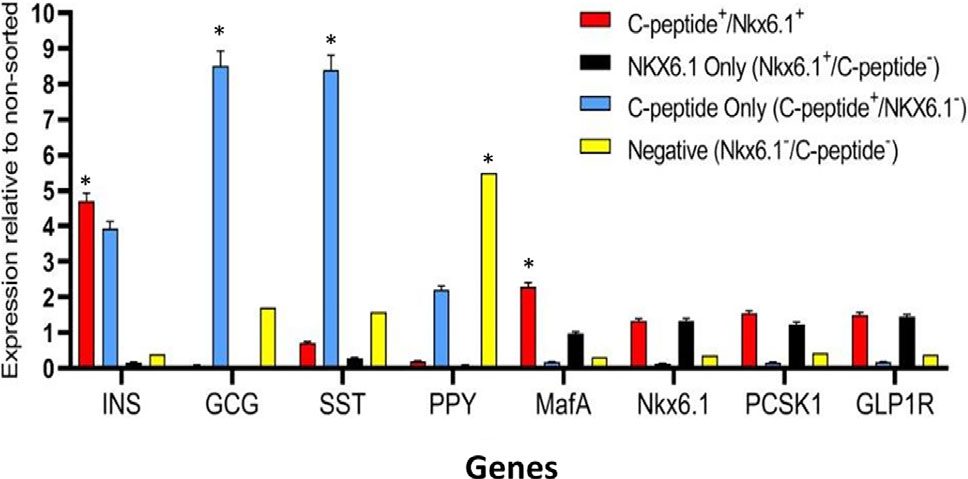
Gene expression analysis of Islet-like clusters stained for C-peptide and NKX6.1. A method for analyzing RNA after intracellular staining (MARIS) was applied to ILC cells. Shortly, dissociated cells were fixed and stained for intracellular C-peptide and NKX6.1 antigens. Following labeling with secondary antibodies, the designated subpopulations were sorted by preparative FACS and RNA from each subpopulation was extracted. The relative expression levels of indicated genes was analyzed by qPCR relative to the non-sorted total ILC cells taken as 1. Results (expressed as mean ± SEM) demonstrated signature genes for specific sorted populations (*significance *p* < 0.05, Student *t* test): Insulin and MAFA is highly expressed in C-peptide**^+^**/Nkx6.1**^+^** population; Glucagon and Somatostatin are highly expressed in C-peptide**^+^**/Nkx6.1**^−^** population (poly-hormonal cells fraction), PPY in C-peptide**^−^**/Nkx6.1**^−^** population. The expression of GLP1R, NKX6.1, and PCSK1 genes is not significantly enriched in one specific population but notably expressed in similar levels in both C-peptide**^+^**/Nkx6.1^+^ and C-peptide^-^/Nkx6.1^+^ populations.

Highly pluripotent hESCs were differentiated according to a seven-stage stepwise protocol carried out in suspension culture conditions (3D), in spinner flasks as well as in controlled bioreactors (as detailed in *Methods*). After stage 7 (days 30–34), 100–200 µm Islet-like clusters (**Figure 1A**) contain hormone-positive cells, especially cells producing insulin (as well as the C-peptide fragment processed from human proinsulin) (**Figure 1B**). These insulin-producing cells typically amount to 60% of the total cells, with about 10% of cells producing glucagon and 2% producing somatostatin (**Figure 1B**). In addition to hormone-producing cells, the hESC-derived ILC still contain precursor cells, since over 90% of cells express the key transcription factor for pancreatic development PDX1 and 70–80% express transcription factors important for β-cell function such as Nkx6.1 and NeuroD1 (**Figure 1C**). Nkx6.1 is of particular importance, being essential for development and function of mature β-cells (12, 13), and serving as a marker of mono-hormonal insulin-producing β-cells (21). In the hESC-derived ILC, these β-cells can be identified by flow cytometry (FACS) as double positive for Nkx6.1 and human C-peptide (**Figure 1D**). This C-peptide^+^/Nkx6.1^+^ double positive fraction usually represents around 20–30% of the total population, the rest being C-peptide^+^/Nkx6.1^−^ (C-pep^+^ only), C-peptide^-^/Nkx6.1^+^ cells (Nkx6.1^+^ only), and C-peptide^−^/Nkx6.1^−^ (Negative, Q3) cells (**Figure 1D**). The four subpopulations were characterized by gene expression (**Figure 2**). After separation by preparative FACS, RNA from each of the fixed and stained cell fractions was extracted and analyzed using the MARIS method (20). The qPCR data relative to the unsorted cells confirmed that the C-peptide^+^/Nkx6.1^+^ double positive compartment is enriched for cells that primarily express insulin. On the other hand, the C-peptide-only fraction is enriched for cells expressing Insulin but also Glucagon (GCG), Somatostatin (SST), and Pancreatic Polypeptide (PPY), identifying these cells with the reported polyhormonal precursors (22–24). Among the four sorted cell fractions, the C-peptide^+^/Nkx6.1^+^ double positive cells had, in addition to insulin, the highest expression level of the transcription factors MafA and Nkx6.1, of the prohormone convertase PCSK1 and the GLP1 receptor (GLP1R) (**Figure 2**). These are characteristics of more mature β-cells. The Nkx6.1-only fraction also showed enrichment for PCSK1, GLP1R, and Nkx6.1 expression, but had lower MafA and very low insulin, suggesting that these are pre-hormonal progenitors. The C-peptide^−^/Nkx6.1^−^ population contained cells expressing GCG, SST, and PPY (**Figure 2**), suggesting the presence of maturing α, δ, and PP islet cells, respectively.

### Identification of CD49A as a Marker for Mature β-Cell

**Figure 3:**
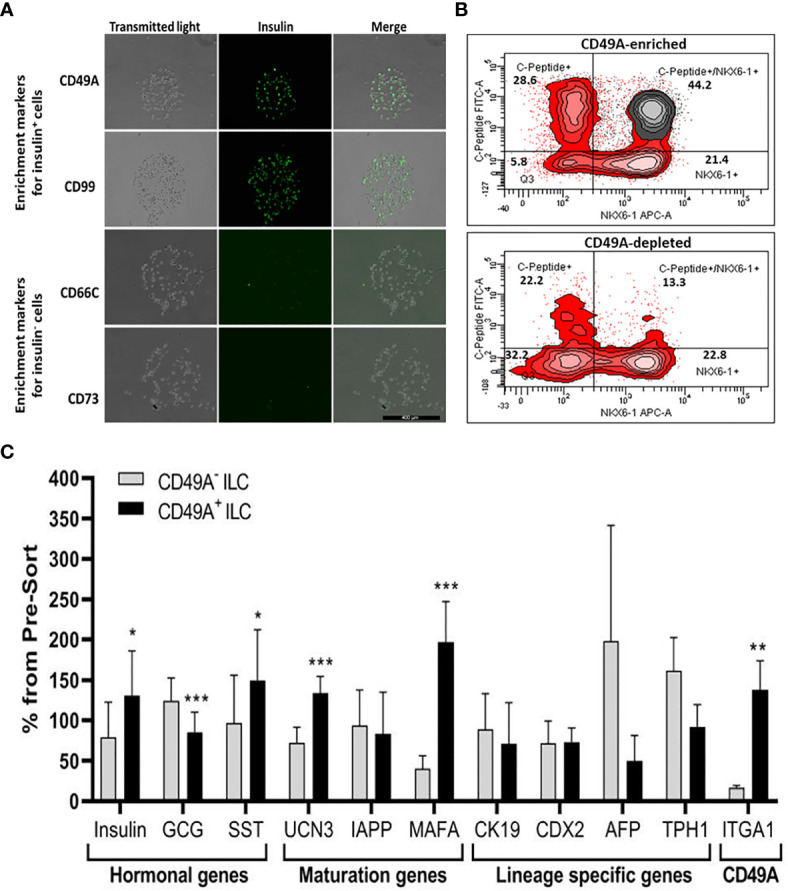
Functional Cell-Capture Screening on antibody array. **(A)** In the array, the spots containing CD49A and CD99 antibodies attach ILC cells that comprise a high proportion of Insulin-positive cells, while spots with CD66c and CD73 antibodies attached cells that do not express insulin. The spots shown were part of arrays of 235 antibodies against cell-surface proteins, reacted with dissociated ILC cells and then stained with anti-insulin antibodies and Cy-5 labeled secondary antibodies. The number of insulin-positive cells was compared to the total cells captured (phase contrast). **(B)** Upper panel: FACS plot of ILC cells selected by MACS for binding to the CD49A antibody (CD49A enriched) and stained for human C-peptide, Nkx6.1, and CD49A, shows that CD49A positive cells (gray) are mainly in the C-peptide^+^/Nkx6.1^+^ double positive fraction. Lower panel: same for fraction that did not bind to the MACS CD49A antibody column (CD49A depleted). For comparative percentage of cells in each FACS subpopulation see **Table 1**. **(C)** qPCR analysis of RNA extracted from ILC cells fractionated by MACS shows that the cells binding to CD49A antibodies (CD49A enriched) have higher Insulin, UCN3, and mainly MAFA expression level. Results calculated in comparison to non-sorted cells, taken as basal 100% reference. The results are expressed as mean ± SEM. Student t test. *p < 0.05; **p < 0.01; ***p < 0.001.

We used the FCCS platform (10) for identifying antibodies to cell surface proteins that preferentially capture insulin-producing cells (**Figure 3A**). Single cells from dissociated ILC were incubated on the array and antibody–bound cells were then stained for insulin. Out of the 235 antibodies in the array, 61 of them captured some ILC cells. Of these antibodies, anti-CD49A consistently captured insulin^+^ cells in amounts exceeding 33% of the total cells captured (the median value for all antibodies being 13%). As illustrated in **Figure 3A**, there were other antibodies binding insulin^+^ cells (e.g. CD99) and several that captured almost only insulin-negative cells (e.g. CD66C, CD73). These latter antibodies may serve for negative selection to remove cells that do not express insulin.

As a marker for positive selection of β-cells, we chose CD49A (Integrin alpha-1) since further experiments showed that this marker is predominantly present in the C-peptide^+^/Nkx6.1^+^ double positive subpopulation (**Figure 3B**, gray in upper panel). Magnetic activated cell sorting (MACS) with antibodies to CD49A, was used to fractionate live ILC cells into CD49A enriched and CD49A depleted populations. A marked increase in the percentage of C-peptide^+^/Nkx6.1^+^ double positive cells was observed by FACS in the CD49A enriched fraction compared to CD49A depleted fraction (**Figure 3B**, lower panel) or to non-sorted ILC cells (**Table 1**, line 1). On the other hand, there was almost no change in the C-peptide-only cells (**Table 1**, line 2). Thus, within the CD49A-enriched fraction, the C-peptide^+^/Nkx6.1^+^ double positive cells became relatively more abundant than the C-peptide-only (i.e. Nkx6.1 negative) and Nkx6.1-only (i.e C-peptide negative) cells. While the proportion of CD49A^+^ cells increased to about 50–60% in the enriched fraction, there were still around 10% of CD49A^+^ cells in the depleted fraction (**Table 1**, line 4). This indicates that even after two passages on a MACS column with anti-CD49A, the separation was not complete. Yet, there may also be heterogeneity in the distribution of CD49A. For example, when the CD49A-enriched fraction was triple stained for CD49A, C-peptide and Nkx6.1, it was found that 81–86% of the C-peptide^+^/Nkx6.1^+^ cells were positive for CD49A (compare lines 5 to line 1 in **Table 1**). However, the same comparison shows that most of the double positive cells remaining in the CD49A-depleted fraction did not express CD49A (only 18–38% of them scoring positive for CD49A). This suggests that the C-peptide^+^/Nkx6.1^+^ double positive population may be heterogeneous, some with and some without CD49A.

**Table 1:** Proportions of C-peptide and Nkx6.1 positive subpopulations in ILC cells selected for binding to CD49A antibodies.

Cell Fraction:	Expt 1			Expt 2		
	Total Non-sorted	CD49A enriched	CD49A depleted	Total Non-sorted	CD49A enriched	CD49A depleted
Cells positive for markers:			Percent of cells			
1. C-peptide/Nkx6.1	17.8	46.2	13.3	22.6	44.2	16.2
2. C-peptide only	24.8	19.7	22.2	25.4	28.6	26.8
3. NKX6.1 only	29.2	10.7	22.8	27.9	15.4	50.4
4. CD49A (overall)	23.8	61.4	9.5	26.7	52.8	10
5. C-peptide/Nkx6.1/CD49A	ND	40.1	5.0	ND	35.9	2.9

**Table 1**. The C-peptide/Nkx6.1 double positive population is increased in the cells binding to CD49A antibodies (CD49A-enriched) compared to cells that did not bind (CD49A depleted). Quantitation of FACS plots (as in **Figure 2B**) of ILC cells either non-sorted or sorted by MACS with anti-CD49a antibody and further reacted with anti-human C-peptide, anti-Nx6.1, and anti-CD49 antibodies. Two different experiments are shown. ND, not determined.

The CD49A-enriched MACS fraction had significant differences in gene expression as compared to the CD49A depleted fraction (**Figure 3C**). After enrichment for CD49A^+^ cells (confirmed by the increase in ITGA1 transcripts encoding CD49A), there was an increase in mRNA for insulin, urocortin-3 (UCN3), and most significantly for MafA. The expression level of glucagon mRNA was somewhat decreased but, unexpectedly, somatostatin mRNA was slightly increased, suggesting enrichment of the relatively small population of δ-cells. Interestingly, transcripts of a hepatic lineage gene (AFP), of which low amounts still remain in the hESC-derived ILC, are further decreased in the CD49A enriched MACS fraction (**Figure 3C**). In addition, TPH1, a gene of the serotonin synthesis pathway, was also reduced (**Figure 3C**), suggesting that CD49A enrichment removes non β-cells producing serotonin inhibiting insulin secretion (25). Overall, the gene expression data confirm that selection for the CD49A cell surface antigen helps to enrich for functional mature β-cells.

In order to be able to transplant cells in large enough amounts, the selection method needs to perform in large-scale preparations. Cells dissociated from ILC at day 35 of differentiation (~200*10^6^ cells), were fractionated by two consecutive passage on MACS columns with anti-CD49A antibodies. The twice retained fraction contained 75 million cells. The quality of this CD49A enriched preparation, evaluated by qPCR, was similar to that of small-scale preparations, with increased Insulin, MafA, and UCN3 mRNA, decreased GCG and AFP mRNAs, relative to non-sorted and CD49A depleted cells. This made it possible to study the *in vivo* activity of the sorted ILC cells to reduce glycemia in diabetes model.

### CD49A Selection Separates ILC Cells That Normalize Glycemia in Diabetic Mice From Inactive Cells

**Figure 4:**
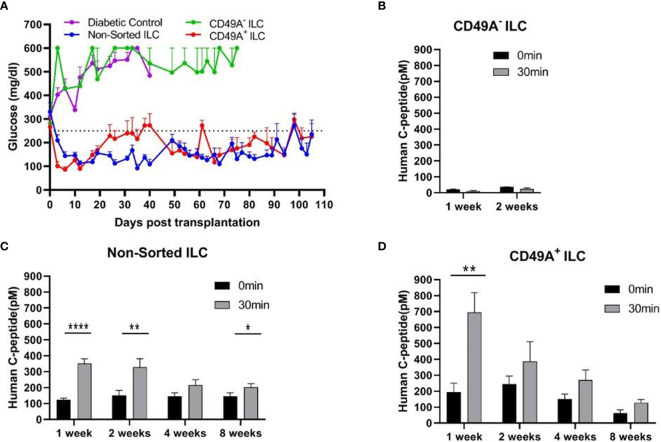
Glycemia of diabetic mice is reduced by CD49A^+^ enriched ILC cells but not by CD49A depleted cells. **(A)** ILC cells at the end of S7 differentiation protocol were fractionated by MACS column with CD49A antibodies. Cells of the bound fraction (CD49A enriched) and of the unbound (CD49A depleted) were reaggregated for 2 days, encapsulated in TMTD-MVG alginate, and implanted by i.p. route (~2*10^6^ cells) into STZ-diabetic mice (n ≥ 5). Non-sorted ILC were similarly implanted. Shortly following transplantation, a reduction of hyperglycemia was observed only in the non-sorted and CD49A^+^ enriched ILC groups, and blood glucose levels of diabetic mice were normalized for more than 100 days. **(B–D)** Glycemia reduction is correlated to human C-peptide glucose dependent secretion. At designated timepoints, mice were tested for human C-peptide concentration levels in blood. Human C-peptide was measured starting from 7 days post-implantation in the three groups of mice after overnight food deprivation (t = 0, black bars) and 30 min after i.p injection of 2 g/kg glucose (t = 30, gray bars). Average blood C-peptide level in pM ± SEM are shown for each group. The glucose-dependent C-peptide levels are significantly higher at day 7 in CD49A^+^ ILC treated mice (p = 0.002), but at later time points, there is no significant functional difference **(C, D)**. The CD49A^−^ ILC treated mice differ significantly from CD49A^+^ enriched and non-sorted treated mice, exhibiting significantly lower levels of human C-peptide **(B)**, suggesting that this ILC population can be depleted (p = 0.034) (Student *t* test). *p < 0.05; **p < 0.01; ***p < 0.001.

Current methods for implantation of human ILC in immunocompetent mice are based on micro-encapsulation in alginate spheres, so as to reduce direct contact of the cells with host immune cells. While the introduction of alginate spheres into the peritoneal cavity of C57BL/6 mice has been shown to elicit foreign body reaction (FBR) and fibrosis even without ILC (8, 9), this reaction can be inhibited by using chemically modified alginate. In particular, long-term functionality was demonstrated for ILC transplants that were encapsulated in low-viscosity SLG20 alginate carrying triazole-thiomorpholine dioxide (TMTD-alginate) and implanted intraperitoneally (i.p.) in mice with streptozotocin (STZ)-induced diabetes (9). In the present study, we used an ultra-pure alginate of medium viscosity (UP MVG alginate) coupled with TMTD for ILC microencapsulation. Microencapsulated ILC, sorted or total, were implanted i.p. into STZ-induced diabetes C57BL/6 mice. The function of non-sorted (Total ILC), CD49A-enriched (CD49A^+^ ILC), and CD49A-depleted (CD49A^-^ ILC) cells was compared by measuring of blood human C-peptide levels and effects on glycemia. Mice implanted with CD49A^−^ ILC fraction cells did not exhibit reduction in BGL levels (**Figure 4A**, purple line), in direct correlation to the low human C-peptide secretion level (**Figure 4B**). Nevertheless, even the low amounts of human insulin secreted improved the viability of CD49A^−^ ILC implanted mice compared to non-implanted diabetic control, which remained highly hyperglycemic and died around day 40 of the follow-up period. The mice implanted with either non-sorted ILC (Blue line, **Figure 4A**) or CD49A^+^ enriched ILC (Red line, **Figure 4A**) exhibited a rapid decrease of blood sugar following implantation, reaching within ~6 days to the normoglycemic range (**Figure 4A**), with the CD49A^+^ enriched ILC exhibited significantly lower BGL values than non-sorted ILC only at the first week. The average non-fasting blood glucose remained mostly in the normal range (below 250 mg/dl) over a follow-up period of 100 days with some fluctuations. These temporal fluctuations can be attributed to changes in ILC composition *in vivo*. The effect on glycemia was confirmed by detection of serum human c-peptide. Albeit CD49A-enriched ILC produced a ~2 fold higher levels of C-peptide than implants of non-sorted ILC at the first week post implantation (**Figures 4C, D**), eventually at later stages, the *in vivo* function results were similar between the groups. Both, non-sorted and CD-49A-enriched, exhibited stimulated human c-peptide secretion 30 min after glucose injection already 1-week post transplantation, demonstrating typical β-cells response to glucose. Overall, these data establish that selection for the CD49A surface marker identifies and separates the ILC cells that control glycemia from inactive ones. On the other hand, the data did not show a clear functional difference between the CD49A-enriched ILC to the non-sorted ILC (**Figure 4A**) during the analysis period. The conclusion from these experiments was that MACS sorting for CD49A by itself was not sufficient to markedly improve the therapeutic activity in the diabetes model, in line with the equivalent levels in the amount of human C-peptide found in the blood of the mice.

### Combining Negative Selection for CD26 With Positive Selection for CD49A

**Figure 5:**
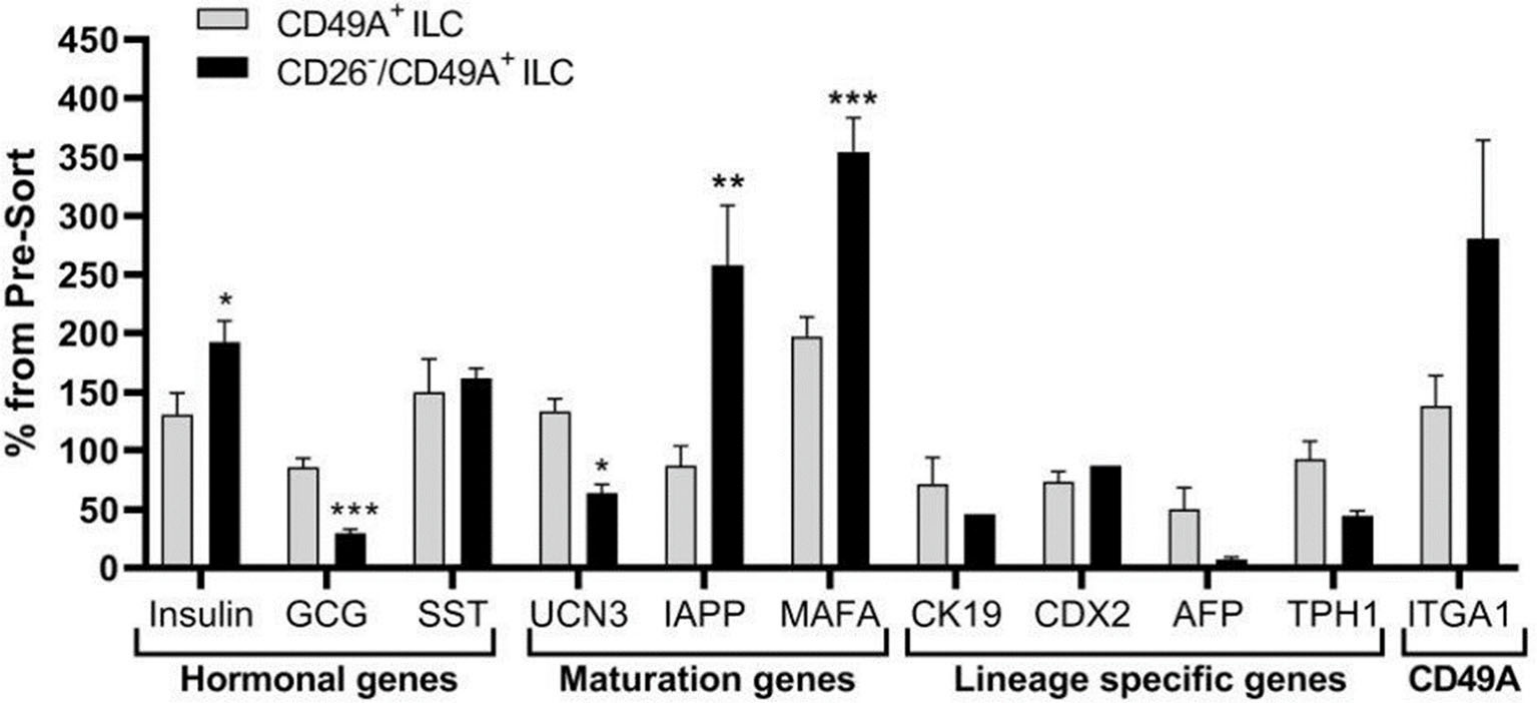
CD26 depletion followed by CD49A selection improves mature β-cells phenotype *in vitro*. qPCR analysis of RNA extracted from ILC cells fractionated by CD26^−^/CD49A^+^ MACS (n ≥ 6 different batches) shows that the depletion/selection strategy yields an improved gene expression phenotype with increased expression of mature islet signature genes as MAFA, Insulin, and IAPP compared to CD49A enrichment alone. In addition, the removal of CD26 results in significantly lower expression levels of Glucagon and AFP mRNAs. Results calculated in comparison to non-sorted cells, taken as basal 100% reference. The results are expressed as mean ± SEM. Student t test. *p < 0.05; **p < 0.01; ***p < 0.001.

**Figure 6:**
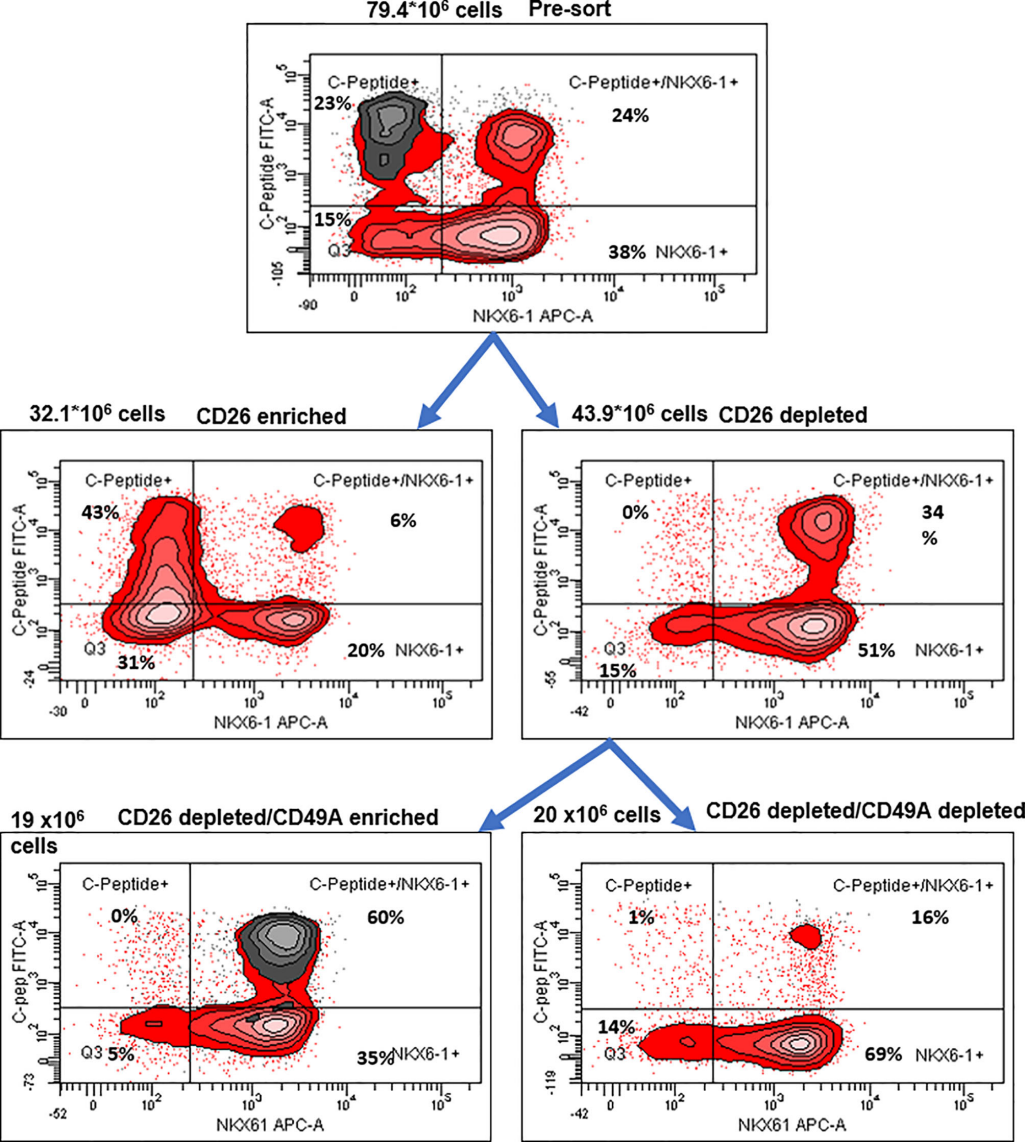
Preparative MACS selection using novel sorting strategy. After selection by MACS with CD26 antibodies (middle two panels), the CD26 depleted cells have lost the C-peptide only fraction (right panel). After subsequent selection of the CD26 depleted fraction by MACS with CD49A antibodies (lower two panels), the C-peptide/Nkx6.1 double positive fraction is increased (lower left panel with CD49A stain in gray). For comparative percentage of cells in each FACS subpopulation see **Table 2**. The total number of cells at each step is shown next to the panels.

Selection for the CD49A marker increases the percentage of C-peptide^+^/Nkx6.1^+^ double positive cells but does not remove the C-peptide-only fraction (**Figure 3B** upper panel and **Table 1**). To improve the selection, we attempted to identify surface markers of ILC cells expressing both Insulin and Nkx6.1 simultaneously. In a new FCCS, each spot of the antibody array was evaluated for capture of insulin^+^ cells and then for the percentage of Nkx6.1^+^ cells among them. We were unable to find an antibody that captured exclusively C-peptide^+^/Nkx6.1^+^ double positive cells, but observed that in the *in vitro* differentiated ILC, an antibody to CD26 (Dipeptidyl peptidase-4, DPP4), captured efficiently insulin^+^ cells (70% of all the captured cells), which only 3.5% of it had Nkx6.1 (**Table S6**). The presence of CD26 on these cells was unexpected since CD26 has been described as a marker of ductal cells and glucagon producing α-cells, but not of insulin-producing β-cells in natural islets isolated from pancreas (26–28). Our finding that in hESC-derived ILC cells, CD26 binds the cells expressing insulin but not Nkx6.1 (**Figure 5**, gray in the uppermost panel) offered the possibility to remove these cells and enrich for cells positive for Nkx6.1 and insulin (or C-peptide). Indeed, MACS selection with anti-CD26 showed that in the unbound cell fraction (CD26 depleted) the C-peptide^+^/Nkx6.1^−^ cells were markedly reduced whereas the C-peptide^+^/Nkx6.1^+^ double positive cells were increased (**Figure 5** and **Table 2**). By a subsequent selection for the CD49A marker (**Figure 5**, lower left panel), the percentage of double positive cells raised and could reach 70% in the CD26 depleted/CD49A enriched fraction (**Table 2** line 1). Gene expression analysis of the CD26^−^/CD49A^+^ ILC cells fraction demonstrated significant differences in gene expression as compared to the CD49A+ enriched population (**Figure 6**). The prior removal of CD26^+^ population resulted in an increase mRNA for insulin, and most significantly for MafA. The expression level of glucagon mRNA was decreased by 70%, and somatostatin mRNA continued to increase, suggesting additional enrichment of the relatively small population of δ-cells. Moreover, depletion of CD26^+^ cells further decreased AFP mRNA levels by ~90% from non-sorted cells (*vs.* 50% decrease by CD49A^+^ enrichment). Overall, the gene expression data confirm that the preparative depletion of CD26^+^ cells from ILC and subsequent selection for the CD49A^+^ cells augment the functional mature β-cell phenotype characteristics.

**Table 2:** Proportions of C-peptide and Nkx6.1 positive subpopulations in ILC cells double selected with antibodies to CD26 and to CD49A.

Expt 1	Total Non-sorted	CD26 enriched		CD26 depleted		CD26 depleted CD49A enriched		CD26 depleted CD49A depleted
Cell fraction								
Cells positive for:	Percent of cells							
1. C-peptide and NKX6.1	24.2	19.7		33.8		71.5		16
2. C-peptide only	22.9	43.0		4.8		10.7		4.0
3. NKX6.1 only	35.8	18.0		52.8		12.1		69.5
4. Negative (Q3)	17.1	19.3		8.5		5.7		10.5
Expt 2								
Cell fraction	Total non-sorted		Total reaggregated	CD26 enriched	CD26 depleted		CD26 depleted CD49A enriched	CD26 depleted CD49A depleted
Cell positive for:	Percent of cells							
1. C-peptide and NKX6.1	22.5		32.6	6.1	33.7		66.6	21.2
2. C-peptide only	20.2		21.7	43.8	8.4		11.4	7.0
3. NKX6.1 only	13.3		21.6	6	24.3		13.3	42.7
4. Negative (Q3)	44.1		24.2	44.1	33.6		8.7	29.1

The C-peptide/Nkx6.1 double positive cells predominate in CD26 depleted/CD49 enriched fraction. After double MACS sorting and staining for human C-peptide and Nkx6.1 cells were analyzed in FACS plots as illustrated in **Figure 5**. Quantitation from fractionation of two independent batches are shown in two experiments. In experiment 2, non-sorted ILC cells after dissociation and reaggregation without fractionation are also shown.

**Figure 7:**
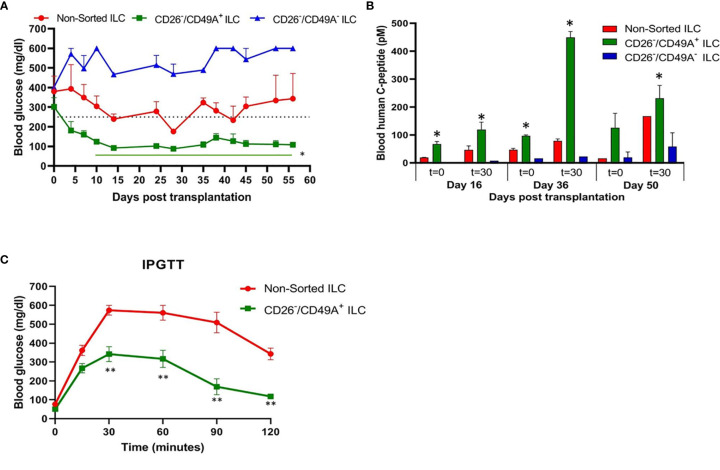
Improved activity of CD26 depleted/CD49A enriched ILC to normalize glycemia in diabetic mice. **(A)** CD26 depleted/CD49A enriched ILC cells (squares with green line) implanted in diabetic C57BL/6 mice, reduce blood glucose better than non-sorted cells (red circles) or than CD26 depleted/CD49A depleted cells (dark blue triangles). After a first MACS with CD26 antibodies, the CD26**^−^** flow-through cells were separated by MACS with CD49A antibodies. The non-sorted and the sorted cell fractions were reaggregated into ILC prior to micro-encapsulation in TMTD-MVG alginate and, from each, a dose of 1 × 10^6^ cells was implanted i.p. in C57BL/6 mice (n ≥ 5). Average blood glucose ± SEM is shown. Starting from day 10, the average blood glucose results of CD26^−^/CD49A^+^ group is significantly lower than non-sorted cells (Student t test. *p < 0.05, horizontal green line). **(B)** In the same mice, blood C-peptide was measured at indicated days, after overnight food deprivation (t = 0) and 30 min after i.p. injection of 2 g/kg glucose (t = 30). Average blood levels of human C-peptide in pM ± SEM are shown for mice with implants of ILC either non-sorted (red), CD26 depleted/CD49A enriched (green), or CD26 depleted/CD49A depleted (blue). The significantly higher values for each timepoint are marked (Student t test. *p < 0.05). **(C)** Intraperitoneal Glucose Tolerance Test (IPGTT) shows that CD26 depleted/CD49A enriched ILC cells (squares with green line) reduce more the diabetic state than non-sorted ILC cells (red circles). On day 45 after implantation the mice were injected i.p with 2 g/kg glucose and blood glucose was measured at different times for a 2-h period. The results are expressed as BGL mean ± SEM. Student t test. **p < 0.01 from t = 30 onwards. The AUC with the CD26 depleted/CD49A enriched cells was 50% lower than that with the non-sorted re-aggregated cells (p = 0.046).

To evaluate their activity for diabetes therapy, implants of CD26-depleted/CD49A-enriched ILC were compared to total non-sorted ILC. We observed that even when the dose of cells implanted was reduced to 1 million cells, the CD26 depleted/CD49A enriched ILC still showed efficiency to rapidly reduce glycemia and maintain it in the normal level (**Figure 7A**, green squares). Under these conditions, the non-sorted ILC did not bring down glycemia to normal levels, but only reduced it slightly and maintained it at an intermediate diabetic state (**Figure 7A**, red circles). The CD49A depleted fraction obtained from the CD26 depleted cells, did not maintain the glycemia, which rose to highly diabetic levels (**Figure 7A**, blue triangles). The levels of human C-peptide in the blood of mice implanted with CD26 depleted/CD49A enriched ILC were higher than with the other types of ILC, throughout the follow-up period of 8 weeks (**Figure 7B**). This was observed in fasting mice and after stimulation of C-peptide secretion by glucose. The increased therapeutic activity of the CD26 depleted/CD49A enriched ILC cells over non-sorted total cells was also demonstrated by an intraperitoneal glucose tolerance test (IPGTT) on day 46 after implantation (**Figure 7C**), as the area under the curve being reduced by 50% (p = 0.046).

## Discussion

Our aim was to fractionate the cell populations produced by differentiation of human pluripotent stem cells into pancreatic islet-like clusters, in order to select the insulin-producing cells which are most active to normalize glycemia in diabetes models. To find markers for selection, we used a functional cell capture screening (FCCS) assay on a microarray of large set of antibodies to cell surface proteins (10). In a first series of screens, we identified an antibody to integrin-alpha1 (CD49A, ITGA1) as efficiently binding insulin-producing cells. We show that by binding to anti-CD49A, one can separate cells that reduce glycemia in diabetic mice from cells completely lacking this capacity, although they express insulin. However, MACS selection with CD49A antibodies was not sufficient to clearly improve the therapeutic activity over that of non-sorted ILC. We, therefore, made a new series of screens in which we compared the antibodies for capture of insulin-producing cells and of Nkx6.1^+^ cells together. We found that an antibody to dipeptidyl peptidase-4 (CD26, DPP4) bound ILC cells expressing insulin (and human C-peptide) but lacking Nkx6.1, and did no bind C-peptide^+^/Nkx6.1^+^ cells (**Figure 5**). By depletion of CD26^+^ cells followed by enrichment for CD49A^+^ cells, ILC populations containing 60–70% C-peptide^+^/Nkx6.1^+^ cells could be obtained. Such purified ILC fractions had an improved efficacy to reduce glycemia and secrete human C-peptide, relative to non-sorted ILC, or cells selected only for CD49A.

CD49A, the Integrin alpha1 chain, had been reported to have a function in the development of β-cells in human fetal and adult pancreas since, in combination with Integrin-β1, it forms the primary collagen-binding receptor (in particular for Collagen IV) and thereby contributes to β-cell adhesion and motility, as well as to insulin secretion (29). In recent studies using single-cell RNA Seq analysis of gene expression (30, 31) in the various cell populations formed by differentiating hPSC toward islet-like cells, Veres et al. (31) found that CD49A (ITGA1) is mostly (but not exclusively) a marker of β-cells expressing the INS, Nkx6.1, NPTX2, PCSK1 genes. These authors reported that MACS with CD49A antibodies can isolate these β-cells at 60–80% purity and that, *in vitro*, glucose-stimulated insulin secretion (GSIS) was improved in these cells. Our approach was different and based on screening of a large array of antibodies against membrane proteins to identify antibodies that bound insulin-producing hESC-derived β-like cells. CD49A emerged from this screen and we proceeded to study the *in vivo* therapeutic activity of cells selected for this marker in models of STZ-induced diabetes in C57BL/6 mice.

CD26 had been reported to be a marker of ductal cells and of alpha cells, but not of β-cells, in islets isolated from human pancreas (26, 27). However, we unexpectedly found that antibodies to CD26 efficiently bound insulin-producing ILC cells lacking Nkx6.1. This finding allowed us to further purify the hESC-derived ILC cells by depletion for CD26 and enrichment for CD49A and thereby obtain ILC with improved anti-diabetic activity in STZ-treated diabetic mice. The correlation between the cell types seen in the selected fractions and the anti-diabetic activity is interesting to investigate. Overall, our data support the assumption that it is the abundance of C-peptide^+^/Nkx6.1^+^ double positive cells which contributes most to the therapeutic activity. In the total non-sorted ILC used in the different experiments, the percentage of double positive cells was in the range of 18–24%, which increased to 34–35% after removal of cells binding to anti-CD26 antibodies and to 60–71% after further purification of the cells binding to CD49A. If the CD49A selection was done alone, only 44–46% double positive were obtained. Hence, the combined CD26 and CD49A selection is better and this is what was observed in term of therapeutic activity in diabetic mice. Similarly, compared to non-sorted cells, the combined CD26 and CD49A selection gave a larger increase in the levels of insulin and MafA mRNA, supporting the effect of enrichment on functional maturation phenotype. Besides enriching for more active β-cells, the purification also removes unnecessary cells. Thus, depleting CD26^+^ cells removed the insulin-producing C-peptide^+^ cells lacking Nkx6.1, which correspond to polyhormonal progenitors that do not seem to contribute to β-cell functions *in vivo* an even to become alpha cells (22) delta cells (13). The beneficial or detrimental role of the other cell types in the ILC remains to be clarified. For example, the CD49A-enrichment also removes cells expressing TPH1 (**Figure 2C**), which probably correspond to cells producing serotonin (entero-chromaffin cells) that inhibit insulin secretion (25). Removing CD26^+^ cells may reduce the amount of DPP4 enzyme which degrades GLP-1, an important stimulator of β-cell function (32). So, removing these populations could eliminate cells that do not participate in the control of glycemia or disturb this control. In addition, and not less important, the purification helps reducing the number of *in vitro* differentiated ILC cells that need to be implanted for cell therapy of diabetes. Our double selection, depleting CD26^+^ cells and enriching for CD49A^+^ cells, removes many cells (**Figure 5**) and allowed to reduce the number of cells needed for normalizing glycemia in our experiments with diabetic mice. This reduction could be of practical importance to decrease the volume of hPSC-derived ILC that will be required to treat human diabetic patients. Performing MACS purification requires dissociation of the differentiated islet-like clusters produced in our 3D-suspension cultures. The dissociated cells, MACS-fractionated or not, were reaggregated by a few days of culture before implantation. This procedure does not alter the composition of the ILC, nor their activity in diabetes models (33). For implantation, we encapsulated the ILC in medium-viscosity MVG alginate coupled with TMTD, and not the very low-viscosity SLG20 alginate as used by Vegas et al. (9). When encapsulated in TMTD-MVG microspheres (of 1.5 mm diameter) and implanted in the peritoneal cavity of the diabetic mice, the total and the purified ILC caused a rapid decrease in glycemia starting soon after implantation. There was no 3–6 month delay as seen when non-mature progenitors are implanted (12). This indicates that the ILC fully differentiated *in vitro*, provide the functionally mature β and other cell types needed to restore and maintain glycemia in the normal range, shortly after implantation. A reservation is that since the MACS fractionation is not complete, the purified fractions are likely to still contain other cell types that influence the therapeutic effect. Indeed, the presence of glucagon-producing alpha-like cells is probably important to prevent hypoglycemic events. The aim is to implant cell clusters that mimic the activities of natural islets, and not pure β-cells. The FCCS assay had previously shown that CD56 and CD9 are markers of β-cell s in islets from human post-mortem donations (11). With our hESC-derived ILC, CD56 was also high on the list of antibodies binding insulin-producing cells, while CD9 was not found (not shown). The CD200 marker reported to isolate endocrine cells at the pancreatic progenitor stage of hESC differentiation (22) was also in our FCCS (not shown). The FCCS assay identified additional antibodies that may be candidates to select cells active in restoring and maintaining normoglycemia in diabetics, and/or remove inactive cells by negative sorting (data not shown). Development of better means of large-scale cell sorting with combinations of antibodies, such as shown here, appears as an important endeavor to produce the amounts of ILC needed for treating the many millions of insulin-dependent diabetic patients by regenerative cell therapy.

## Conclusions

*In vitro* differentiation of islet-like clusters from human pluripotent stem cells represents a potentially unlimited source of cells that could restore physiological control of blood glucose in diabetic patients requiring insulin. The CD49A surface protein (integrin-alpha1) was identified as a selective marker of the ILC cells that are active to normalize glycemia in a diabetic mice model. Removal of cells expressing CD26 (DPP4) prior to enrichment of CD49A^+^ cells further improved their therapeutic activity and reduced the number of ILC cells needed to normalize glycemia. The ILC purification described appears as a promising strategy to improve cell therapy of diabetes with hPSC-derived ILC.

## Data Availability Statement

The original contributions presented in the study are included in the article/**Supplementary Material**. Further inquiries can be directed to the corresponding author.

## Ethics Statement

The animal study was reviewed and approved by National Council for Animal Experimentation, MOH, Israel.

## Author Contributions

KM, JC and MR directed the research work and wrote the manuscript. OE, MW and YS conceived and contributed the Functional Cell Capture Screening. DB, AB, KY, MZ, IT, AE and EV performed the experiments. AL, KM supervised the animal experimentation. AH and JI were senior advisors. All authors contributed to the article and approved the submitted version.

## Funding

This work was supported by the Israel Innovation Authority grants No. 51743, 54659, and 56956.

## Conflict of Interest

KM, DB, AB, KY, MZ, IT, AE, AL, AH, JI-E, and JC are researchers employed in the Biotechnology Company Kadimastem Ltd., Nes Ziona, Israel. MR is a major shareholder of Kadimastem.

The remaining authors declare that the research was conducted in the absence of any commercial or financial relationships that could be construed as a potential conflict of interest.

## Abbreviations

AFP, alpha fetoprotein; BSA, bovine serum albumin; CMRL, CMRL serum free medium; DPP4, dipeptidyl peptidase-4; ES, embryonic stem; FACS, fluorescent activated cell sorting; FBS, fetal bovine serum; FCCS, functional cell capture screening; GCG, glucagon; GLP1R, glucagon-like peptide-1 receptor; GSIS, glucose stimulated insulin secretion; HPRT, hypoxanthine phosphoribosyl transferase; hPSC, human pluripotent stem cell; hESC, human embryonic stem cell; IAPP, islet amyloid polypeptide; ILC, islet-like cluster; ip, intraperitoneal; IPGTT, intraperitoneal glucose tolerance test; ITGA1, integrin alpha1; MACS, magnetic activated cell sorting; MafA, Pancreatic B-Cell-Specific Transcriptional Activator; Nkx6.1, Nk6 homeobox-1;NMR, nuclear magnetic resonance; NPTX2, neuronal pentraxin-2; PBS^−/−^, phosphate buffered saline, no calcium, no magnesium; PCSK1, prohormone convertase; PFA, paraformaldehyde; PPY, pancreatic polypeptide Y; qPCR, quantitative Polymerase Chain Reaction; RT, room temperature; SST, somatostatin; STZ, streptozotocin; TMTD, triazole-thiomorpholine-dioxide; TPH1, tryptophan hydroxylase-1; UCN3, urocortin-3.
