# Supplementary material for: Selection for CD26− and CD49A+ Cells From Pluripotent Stem Cells-Derived Islet-Like Clusters Improves Therapeutic Activity in Diabetic Mice
Source: Front Endocrinol (Lausanne). 2021 May 5;12:635405. doi: 10.3389/fendo.2021.635405 (PMC8131825; doi:10.3389/fendo.2021.635405)
Supplement: Supplementary file 1 [file DataSheet_1.docx]

**Additional file 1.**

**Table S1**. **Additives to basal medium in the course of the seven-stage differentiation (30 to 44 days).**

|  |  | **BASAL** |  |  |  |  |
| --- | --- | --- | --- | --- | --- | --- |
| **STAGES** | **DAY** | **MEDIUM** | **FACTORS** | **CONC.** | **COMPANY** | **CATALOGUE** |
| Stage 1 | 0 | A | Activin A | 100ng/ml | Peprotech | 120-14 |
|  |  |  | CHIR 99021 | 3uM | CAYMAN | 13122 |
|  | 1 | A | Activin A | 100ng/ml | Peprotech | 120-14 |
| Stage 2 | 3 to 5 | B | KGF | 50ng/ml | Peprotech | 100-19 |
| Stage 3 | 6 to 7 | C | KGF | 50ng/ml | Peprotech | 100-19 |
|  |  |  | SANT-1 | 0.25uM | SIGMA | S4572 |
|  |  |  | RA | 1uM | SIGMA | R2625 |
|  |  |  | TPB | 200nM | Calbiochem | 565740 |
|  |  |  | LDN 193189 | 100nM | Biological industries | 04-0074 |
| Stage 4 | 8 to 11 | D | KGF | 50ng/ml | Peprotech | 100-19 |
|  |  |  | SANT-1 | 0.25uM | SIGMA | S4572 |
|  |  |  | RA | 0.1uM | SIGMA | R2625 |
|  |  |  | TPB | 100nM | Calbiochem | 565740 |
|  |  |  | LDN 193189 | 200nM | Biological industries | 04-0074 |
| Stage 5 | 12 to 14 | E | SANT-1 | 0.25uM | SIGMA | S4572 |
|  |  |  | RA | 0.05uM | SIGMA | R2625 |
|  |  |  | Alk5i II | 10uM | CAYMAN | 14794 |
|  |  |  | T3 | 1uM | SIGMA | T6397 |
|  |  |  | Zn SO4 | 10uM | SIGMA | Z0251 |
|  |  |  | LDN 193189 | 100nM | Biological industries | 04-0074 |
| Stage 6 | 15 to 22 | F | Alk5i II | 10uM | CAYMAN | 14794 |
|  |  |  | T3 | 1uM | SIGMA | T6397 |
|  |  |  | Zn SO4 | 10uM | SIGMA | Z0251 |
|  |  |  | GSiXX | 0.1uM | SIGMA | SML0649 |
|  |  |  | LDN 193189 | 100nM | Biological industries | 04-0074 |
| Stage 7 | 23 to 29 | F | Alk5i II | 10uM | CAYMAN | 14794 |
|  |  |  | T3 | 1uM | SIGMA | T6397 |
|  |  |  | Zn SO4 | 10uM | SIGMA | Z0251 |
|  |  |  | ALXi-R426 | 2uM | APExBIO | A8329 |
|  |  |  | Trolox | 10uM | CAYMAN | 10011659 |
|  |  |  | N-Acetyl Cysteine | 1mM | SIGMA | A9165 |
|  |  |  | Dexamethasone | 100nM | SIGMA | D4902 |
| Stage 8 | 30-44 | CMRL^+^ |  |  |  |  |
|  |  |  |  |  |  |  |
|  |  |  |  |  |  |  |

**Table S2**. **Basal media composition and indicated additions to one liter during differentiation**

|  | Day0-3 | Day 4-5 | Day 6 | Day 7-11 | Day12-30 | Day30-40 | Company | Cat. Number |
| --- | --- | --- | --- | --- | --- | --- | --- | --- |
| BASAL MEDIUM | A | B | C | D | E | CMRL+ |  |  |
| MCDB (L) | 0.97 | 0.97 | 0.97 | 0.97 | 0.97 | 0 | Gibco | 10372019 |
| Glucose (g) | 0.45 | 0.45 | 0.45 | 0.45 | 2.5 | 0 | SIGMA | G8769 |
| NaHCO3 (g) | 2.46 | 1.754 | 1.754 | 1.24 | 1.754 | 0 | SIGMA | S5761 |
| FAF BSA (g) | 5 | 5 | 20 | 20 | 20 | 20 | Proliant | 68700 |
| ITS-X (ml) | 0.02 | 0.02 | 5 | 5 | 5 | 0 | Gibco | 51500056 |
| Glutamax (ml) | 10 | 10 | 10 | 10 | 10 | 0 | Gibco | 35050038 |
| P/S (ml) | 10 | 10 | 10 | 10 | 10 | 10 | Gibco | 15140-122 |
| Vit C (mg) | 44 | 44 | 44 | 44 | 44 | 0 | SIGMA | A5960 |
| Heparin (mg) | 0 | 0 | 0 | 0 | 10 | 0 | SIGMA | H3149 |
| CMRL (L) | 0 | 0 | 0 | 0 | 0 | 1 | Biological industries | 01-821-1A |

|  |  |
| --- | --- |

**Table S3.** **List of the qPCR primers**.

|  | ABI | IDT |
| --- | --- | --- |
| Nkx6.1 |  | Hs.PT.58.25073618 |
| HPRT | HS99999909 _ m1 |  |
| Glucagon |  | HS.PT.58.14706508 |
| AFP | Hs00173490 _ m1 |  |
| Rfx6 | Hs00543100 _ m1 |  |
| Ngn3 |  | Hs.PT.53a.19734677g |
| Sox9 |  | Hs.PT.58.38984663 |
| Cdx2 | HS01078080 _ m1 |  |
| TBP | Hs00427621 _ m1 |  |
| MAFA  Insulin | Hs02741908_m1 | HS.PT.58.14570025.g |

**MARIS protocol: Method for Analyzing RNA following Intracellular Sorting**

The MARIS procedure was used as described (18) to compare gene expression in four populations of cells sorted from hESC-derived islet-like clusters. Prior to sorting, the cell clusters were dissociated with TrypLE Express (Invitrogen #12604), and the enzyme neutralized by addition of PBS with 10% FCS. After counting, cells were washed with PBS and suspended in PBS 1x, (made in RNase and DNase-free water) and containing RNase inhibitor (Promega N2615), containing 4% PFA. After incubation at 4^o^C for 20 minutes, cells were washed twice in PBS and suspended in 100 μl of blocking buffer (5% BSA, 3% horse serum) containing 0.3% Triton-X100, and incubated for 1h at 4^o^C. After centrifugation, cells were suspended in blocking buffer 0.1% Triton-X100, with RNase inhibitor, anti NKx6.1 PE or APC and anti-human C-peptide (RAT, DSHB), then incubated for 1h at RT in the dark. After washing with PBS, the pellet was suspended in blocking buffer containing 0.1 μl of donkey anti-rat 488 (Jackson), RNase inhibitor and incubated for 1 hour in the dark. After washing with PBS and centrifugation at 1000xg for 3 min, cells were suspended in FACS buffer for sorting. Four distinct populations were sorted: C-peptide only; C-peptide**^+^** and Nkx6.1**^+^** (DP, double positive), Nkx6.1 only; Q3 negative for C-peptide and Nkx6.1. Cells with highest fluorescence intensity of each population were taken, with an efficiency of 75%. Cells were collected in PBS containing RNase inhibitor and RNA extracted with the RNeasy FFPE Kit (AMBION- recover all total nucleic acid), and treated with DNase before RNA recovery. RNA was converted to cDNA and gene expression evaluated by qPCR.

**Table S4**. **List of antibodies used**.

| **Antibody** | **Company** | **Cat. Number** |
| --- | --- | --- |
| Alexa flour 647 mouse anti-human Nkx6.1 | Becton Dickinson bioscience | 563338 |
| PE mouse anti-human PDX-1 | Becton Dickinson bioscience | 562161 |
| PE mouse anti-human NeuroD1 | Becton Dickinson bioscience | 563001 |
| PE mouse anti-human CD49a | Miltenyi Biotech | 130-101-397 |
| Rat monoclonal anti-human C-peptide | DSHB | GN-ID4-c |
| Goat anti-human PDX1 | R&D systems | AF2419 |
| Mouse anti-human Glucagon | Santa Cruz | Sc-514592 |
| Goat anti-human Glucagon | Santa Cruz | Sc-7780 |
| Rabbit anti-human Somatostatin | Abcam | AB11765 |
| Alexa flour 488 Donkey anti-rat IgG | Jackson immune research | 712-545-153 |
| Alexa flour 647 Donkey anti-mouse IgG | Jackson immune research | 715-605-151 |
| CY3 Donkey anti-goat IgG | Jackson immune research | 705-165-147 |
| Alexa flour 488 Donkey anti-goat IgG | Jackson immune research | 705-545-147 |
| Alexa flour 647 Donkey anti-rabbit IgG | Jackson immune research | 711-605-152 |
| Anti-PE microbeads | Miltenyi Biotech | 130-048-801 |

**Table S5**. **CD26 and other markers identified by FCCS**

| Antibody to | Insulin-positive in percent of PDX1-positive cells | Nkx6.1-positive in percent of insulin-positive cells |
| --- | --- | --- |
| CD49A | 37 | 24 |
| CD26 | 70 | 3 |
| CD99 | 19 | 8 |
| CD200 | 50 | 6 |
| CD56 | 30 | 5 |
| CD66c | 1 | 0 |
| CD73 | 1 | 0 |

Dissociated ILC cells were spread on arrays of 233 antibodies against cell-surface proteins and after incubation the cells bound to each spot on the assay were reacted with anti-PDX1, anti-insulin and anti-Nkx6.1 antibodies. The proportion of cells stained for each antigen in the total cell captured was evaluated.


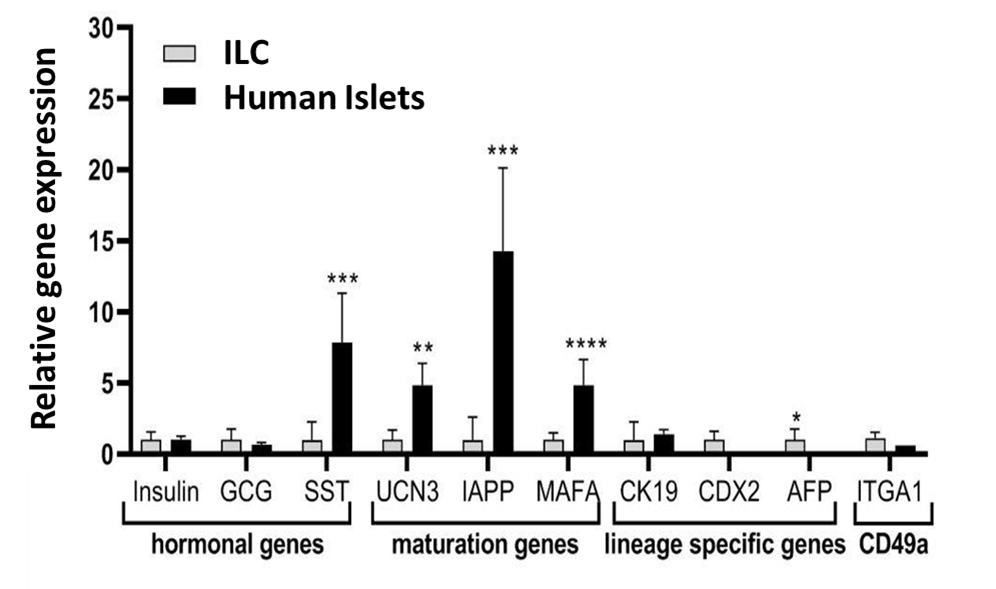
**Figure S1. Relative expression of non-sorted ILCs in comparison to donor human islets**

The expression levels of indicated genes in human islets was analyzed by qPCR relative to the non-sorted total ILC cells taken as 1. Results (expressed as mean ± SEM) demonstrated that in comparison comparison to human islets, hESC-derived ILC cells express significantly lower levels of SST, UCN3, IAPP and MAFA. AFP is highly expressed, while no significant difference was found relative to glucagon, CK19, CDX2 and CD49a expression.

**Figure S2.** **Flow cytometry analysis for NeuroD1 and CHGA expression**


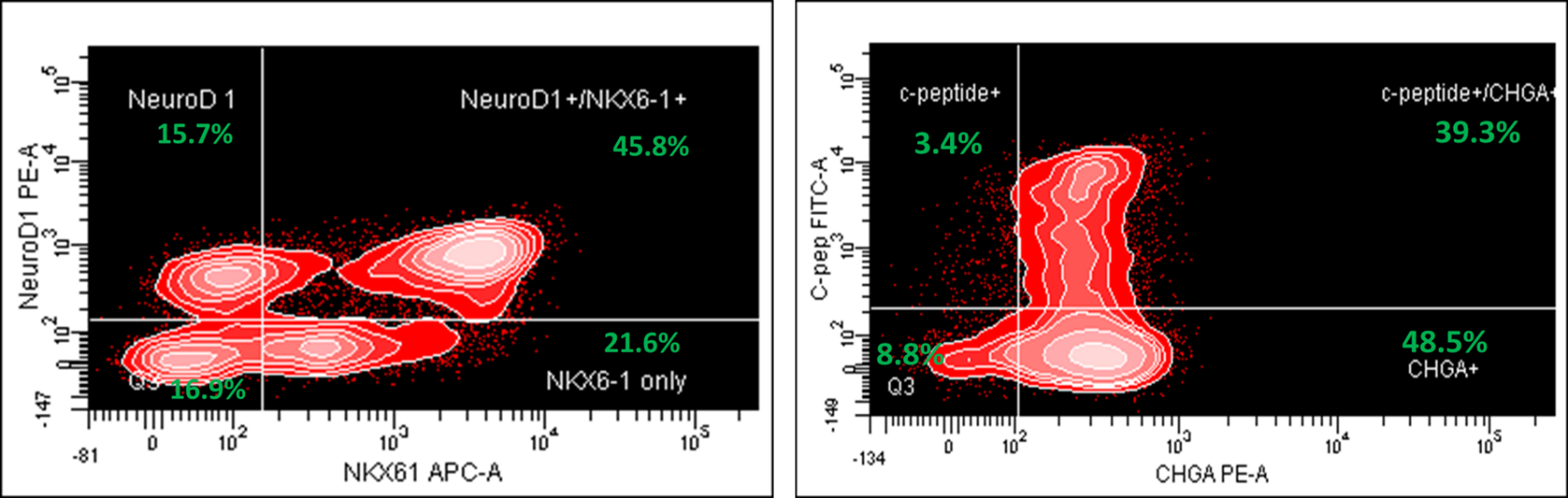


Flow-cytometry analysis of dissociated total ILC cells, fixed and stained for NeuroD1 and Nkx6.1 (left panel) and c-peptide and CHGA (Chromogranin A), (right panel). The percentage of each designated population is shown in quadrants (highlighted in green).

**Figure S3. Immunofluorescence of ILC cells for somatostatin expression**


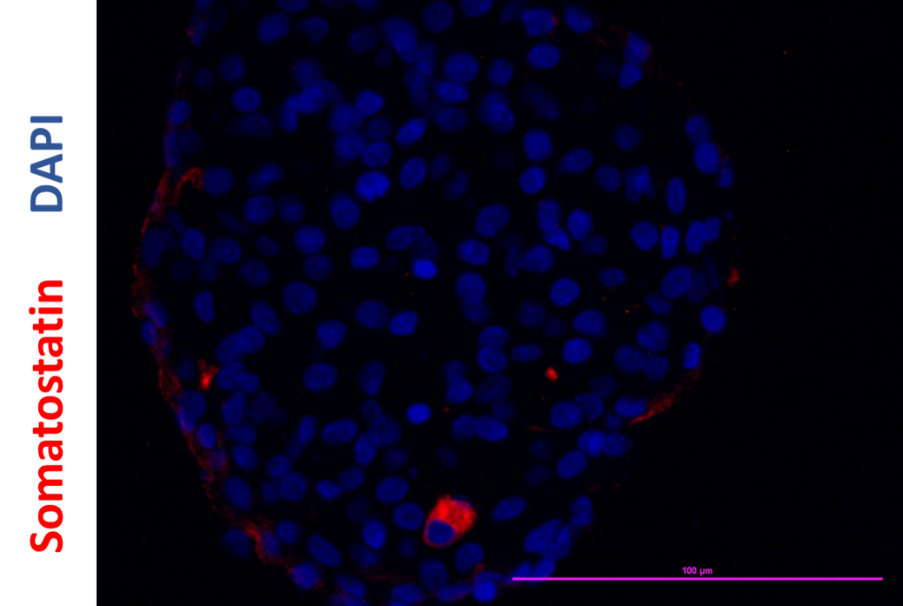


Immunostaining of an ILC for Somatostatin expression (red). The percentage of Somatostatin cells is low, ~1%. Nuclei is counterstained with DAPI (blue).

**Figure S4.** **Immunofluorescence of microencapsulated ILC cells explanted from mice ~6 weeks post transplantation.**


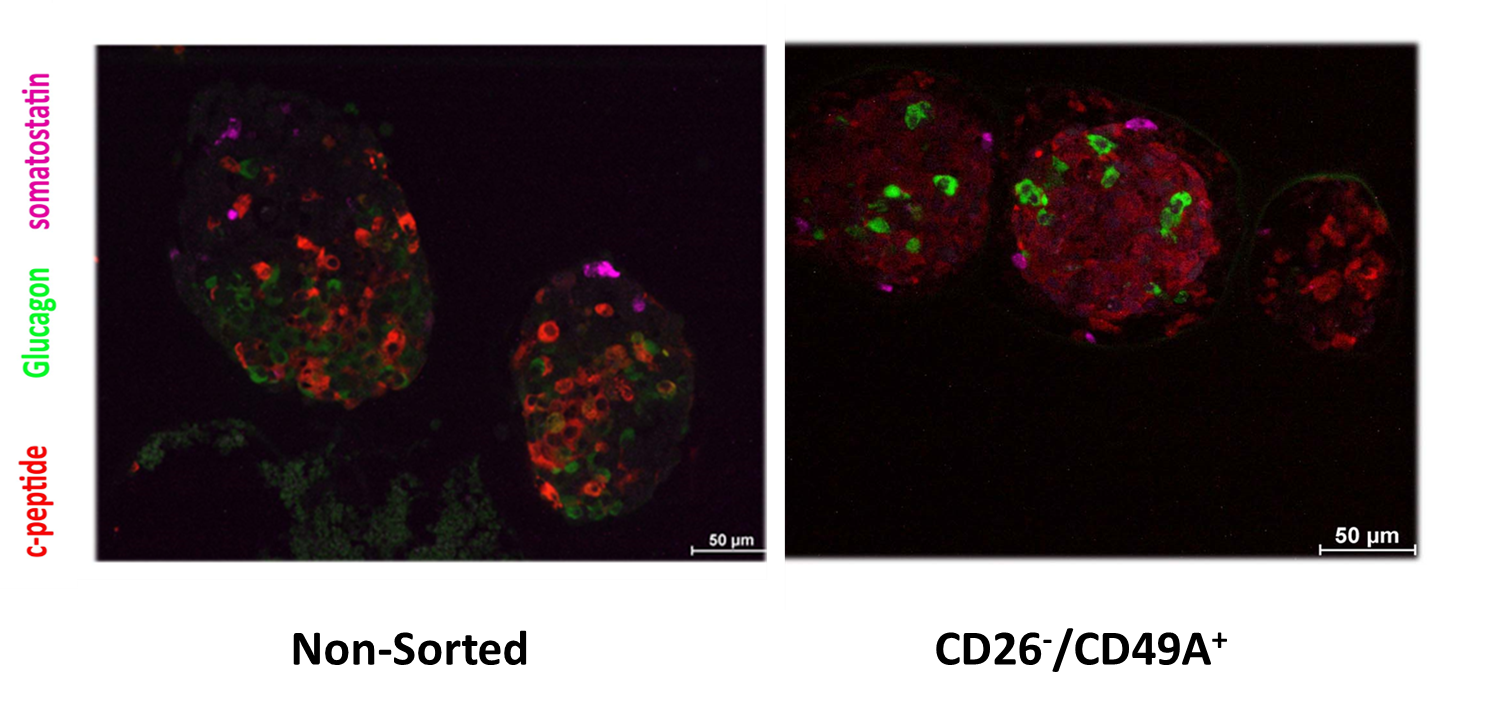


Immunostaining of explanted microencapsulated ILC following 6 weeks in vivo for c-peptide (red), Glucagon (green) and Somatostatin (purple) expression. ILC sorted by CD26^-^/CD49A^+^ combination exhibit homogenous insulin positive cells relative to non-sorted ILC cells.
